# Summary of the Transactions of the College of Physicians of Philadelphia

**Published:** 1847-01

**Authors:** 


ART. V.—*Summary of the Transactions of the College of Physicians of Philadelphia. From Sept. to Nov.*, 1846, *inclusive.*

We always greet with pleasure this quarterly publication, and we would again express the hope that similar associations in other cities will follow the commendable example of the College of Physicians in Philadelphia, not only in holding stated monthly meetings for reports, discussions, and the communication of individual experience, but in the regular publication of their proceedings, for the benefit of the profession at large.

The last number of the Summary contains an interesting report by Dr. Samuel Jackson, formerly of Northumberland, Pa., on the *Theory and Practice of Medicine*, to which we cannot do justice by extracts, and the length of the article precludes its entire insertion. The reporter devotes considerable space to critical remarks upon the review of Homœopathy by Dr. Forbes, which has excited so much discussion of late, both within and without the walks of medical literature. Dr. J. holds a pungent pen, which does not permit Homœopathy and its involuntary defender Dr. F., to escape unscathed. Were our limits more extended we should be glad to give quotations of this portion of the report for the sake of the writer's lively, piquant style; the subject we deem of too little importance to claim much attention. We must still hold to our opinion that if people will be fools they must be indulged, or rather, it is useless to attempt to reason them out of their folly. They must work their own reformation from their own experience; and fortunate are they if conviction does not come too late! Dr. J. next controverts the proposition "*that quackery is more and more favored by the people, and science condemned.*" We have long deemed the common belief in this proposition to be incorrect. From the earliest history of medicine, in every age, the prudent and sagacious have found abundant occasion to lament a prevailing tendency to imbibe and encourage medical delusions. We do not believe this tendency to be on the increase; but, at the same time, it must be admitted, that as science and civilization advance, there is less apology for the success of quackery. The world manifestly, in this respect, does not grow wiser in proportion as it grows older.

Lastly the writer examines the foundation of the doctrine that "*Medicine is an uncertain Science,*" and shows that while it is partly true, the concession has given rise to great abuses.

The "Transactions" also contains elaborate reports on vaccination and the variolous epidemic. The latter consists chiefly of replies from a large number of practical physicians to certain interrogatories propounded by the committee. They present considerable discrepancy of experience. The following are the general deductions :

*First*—That children, or persons under the age of puberty, rarely take the re-vaccination, if the previous vaccination has been perfect. All the cases reported furnishing but one unequivocal case of successful re-vaccination, and that in a constitution peculiarly susceptible.

*Secondly*—That though there is a great disparity in some of the reports, yet the large proportion of the respondents agree in the opinion, that vaccination in an adult, once protected by previous vaccination, inoculation, or an attack of small-pox, will, in some instances, pursue the course, and present the characteristic appearances of the genuine disease. The proportion of cases in which this result will take place cannot be definitely arrived at from the materials before us.

*Thirdly*—It is evident, from the tenor of some of the replies, that those physicians who have reported the highest rates of successful cases, have fixed in their minds a standard of what constitutes a perfect pock much lower than that which is held by the great mass of the medical profession; and, on the other hand, that those gentlemen who, in a large number of re-vaccinated cases, have seen no case which they would deem perfect, have an estimate of the perfect pock which the public sentiment of profession, judging from the replies now before us, and from the published reports on the same subject from Europe, would not sustain.

The Committee consisted of Isaac Parish, Henry Bond, and John Rodman Paul. The report on the "*Protective powers of Vaccination*," exhibits the results of an elaborate examination of the subject, and extensive experimental observations. We quote the concluding portion of the report, which presents a numerical account of the phenomena following re-vaccination in a large number of cases, and the final conclusions of the committee.

The second question, therefore, submitted to your committee, was to determine the phenomena resulting when those who have been already vaccinated are again subjected to the disease, and the necessity and policy of re-vaccination. It is to the first portion of this question that the committee have been obliged, necessarily to confine their attention. It must be very evident that to test fully the necessity or propriety of re-vaccination, would either require that after large communities shall have been a second time subjected to vaccination, time be allowed in order that they may be repeatedly subjected to the influence of variolous contagion, during the epidemical visitations of small-pox, or that the direct test be resorted to of inocculating with small-pox those who have been successfully vaccinated, at different periods subsequent to the primary operation, as well as after a secondary operation, and carefully noting the comparative immunity from the impression of the variolous poison exhibited by each class of patients.

In order to obtain as wide a field of observation as was in the power of your committee, they applied to the comptrollers of the public schools, soon after their appointment, for the privilege of vaccinating the scholars whose parents would consent to the operation being performed, whether they had been previously vaccinated or not, in order to ascertain the degree of impression that would be made in each case by a repetition of the operation, and with a view of watching hereafter the effect or influence it may have in preventing the occurrence of small-pox in the subjects of our experiments, should an epidemic of that disease again appear amongst us. But the comptrollers did not at that time consent to our request, and the investigation of the subject had to be postponed. In the early part of the present year, small-pox again made its appearance, and excited considerable uneasiness in the minds of the public. Your committee entertaining the opinion that the period was propitious for a renewal of their application to the comptrollers, accordingly did so, and their consent was promptly given. Circulars were then sent to the parents of the children attending several of the schools, requesting their permission, which was in most cases readily granted, and every facility was offered to carry into effect the object your committee had in view.

The operation was performed in nine hundred and thirty-one cases; six hundred and thirty-five of whom took on various grades of action; many of these had been vaccinated three, four and five times before, and stood in relation to small-pox, chicken-pox, and varioloid as stated in the following account :—

Nine hundred and thirty-five persons reputed to have been protected by previous vaccination, having distinct cicatrices on the arm, or by having passed through the small pox, were subjected to the action of the vaccine virus.

50 did not appear on the days appointed for examination.
294 exhibited no signs of inflammation from the process.

| | | | | |
|---|---|---|---|---|
| 52 | of these | were reputed to have been previously vaccinated | | twice. |
| 6 | " | " | " | three times. |
| 1 | " | " | " | four times. |
| 14 | " | to have had the small-pox. | | |
| 35 | " | " | varioloid. | |
| 72 | " | " | chicken-pox. | |

76 had various degrees of inflammation on the fourth day, which had faded before the eight day.

| | | | | |
|---|---|---|---|---|
| 15 | of these | were reported to have been previously vaccinated twice. | | |
| 2 | " | " | to have had the small-pox. | |
| 2 | " | " | " | varioloid. |
| 26 | " | " | " | chicken-pox. |

181 had inflammation more or less severe, accompanied by a yellow or purulent crust, on the eight day.
30 of these were reported to have been previously vaccinated twice.
11 " " " three times.
2 " " " four times.
1 " " " seven times.
9 " to have had the small-pox.
7 " " " varioloid.
50 " " " chicken-pox.
95 had very diffusive inflammation, the pustule still moist on the 8th day.
10 of these were reported to have been previously vaccinated twice.
1 " " " three times.
1 " " " four times.
2 " to have had the small-pox.
4 " " varioloid.
23 " " chicken-pox.
179 exhibited more or less inflammation, and a brown crust on the 8th day.
10 of these were reported to have been previously vaccinated twice.
4 " " " three times.
1 " " " seven times.
8 " to have had the small-pox.
7 " " varioloid.
49 " " chicken-pox.
57 had a pock with various degrees of inflammation; diffused and irregular in its margin on the 8th day.
2 of these were reported to have had the small-pox.
16 " " chicken-pox.
5, viz: a boy and two girls, exhibited a well-defined pock and the areola characteristic of the perfect vaccine impression as observed on the 8th day. They are stated not to have been affected with the small-pox, varioloid, or chicken-pox.

From the foregoing observations, as well as from those derived from previous practice, your committee are justified in the conclusion, 1st. that vaccination is the best preservative of human life now known against the contagion of small-pox; and although it has not protected the system in all instances againt at least a modified form of variola, which is the case with small-pox itself, nevertheless life is very generally protected by it, and humanity and sound practice imperiously call for its continuance.

2ndly. Patients who have been once fully subjected to the influences of the vaccine infection, lose, in a great degree, their susceptibility to a second infection; the susceptibility diminishing still more at every subsequent vaccination.

3rdly. That portion of the community who have been once successfully vaccinated, are, in the great majority of cases, fully protected from small-pox, or varioloid.

4thly. A second vaccination does not insure the system in every instance against an attack of varioloid; neither does second vaccination prevent an impression being made on the system by a subsequent operation.

5thly. Upon the occurrence of small-pox in a family or neighborhood, it is important that all individuals in regard to whom there is any doubt or uncertainty as to the fact of their having been successfully vaccinated, should be subjected immediately to the operation, this being the most certain means of preventing the spread of the variolous contagion.

Your committee have not heard of a single instance of varioloid having occurred in any of the children of the public schools vaccinated by them, notwithstanding the epidemic was prevailing at the time and subsequently.

D. Francis Condie,

Thomas T. Hewson, } *Committee.*

J. Wilson Moore.

Accompanying this report there was presented a table containing the name and age of each individual re-vaccinated by the committee, the number of times each had been previously vaccinated; noting those who had been affected with small-pox, varioloid, or chicken-pox; and the appearance of the arm on the fourth and eighth and twelfth days, with remarks on the result of each case.

In conclusion we give some interesting remarks on the *Treatment of Dysentery* by Dr. Huston.

Dr. Huston remarked, that he had, unfortunately, a great deal of experience in the treatment of dysentery, having in early life practiced in a district of country where, during several seasons, it prevailed extensively; he had suffered five attacks of the disease himself, in several of which he had been extremely ill.

In the section of country where he had formerly resided, (near Chester, in this State,) the disease generally broke out in spots and spread amongst some four or five families in a particular neighborhood, appearing to be communicated by contagion. The disease in this form was marked by violent symptoms, there was an almost constant desire to go to stool, with excruciating pain, causing strong men to cry out with the suffering; there was intense thirst in many instances; in some cases petechiæ, stools resembling bloody water or meat scrapings were not uncommon; he generally lost some patients, and sometimes two or three in one family. The practice pursued by him in these epidemics was to give calomel, opium, and ipecac., with castor oil and laudanum, injections, and emollient drinks.

Recently, however, Dr. Huston relied mainly on opium in the treatment of dysentery; he seldom gives calomel, and then only in cases where the tongue is furred. His rule is, to give the opium in sufficient doses to stop the pain—nothing exhausts the system like pain—if restricted to blood-letting or opium in the treatment of all abdominal inflammations, he would unhesitatingly take the opium; he has no fear of its stimulating properties in these diseases when given in sufficiently decided doses. In addition to the opium, the patient should be kept quiet, and food should be selected which will not produce much fæces. If the stomach be irritable, the opium may be given in enemeta, if not, by the mouth or enemeta, as circumstances may dictate. He generally begins the treatment by giving 2 grs. to an adult, and this dose is repeated until the influence of the remedy is felt, when the quantity is diminished; his rule is to give the opium immediately after a stool, especially if it be given in the form of enema. Dr. Huston has laid aside the use of ipecac. in this disease, from its tendency to purge; when combined with the opium, even in the dose of ½ gr. it will frequently purge; this fact he has repeatedly tested to his satisfaction. The ipecac. has also a tendency to produce nausea and vomiting, even in small doses, effects which it is desirable to avoid in this disease. In some cases he has prescribed 15 drops of laudanum after each stool, with the happiest effect; relief to the pain, and free diaphoresis being produced. The manner in which opium acts in this disease is, besides its constitutional effect upon the nervous and vascular systems, by controlling the contraction of the muscular coat of the bowels, which is the great source of pain and danger. Since pursuing the opium practice, Dr. H. has had much less trouble in the treatment of dysentery; cases generally got well in a week, and he has seen few fatal cases. How this treatment might succeed in the more violent forms of epidemic dysentery occurring in country situations, Dr. H. will not pretend to assert; if he were to encounter such cases again he would certainly meet them with less fear than formerly, in the confidence that this course of treatment would be more effectual than that which he had previously adopted.

In regard to the use of castor oil, Dr. Huston remarked, that he was in the practice of giving this article in the dose of a teaspoonful every two or three days, for the purpose of producing a slight purgative effect and thus of keeping the bowels clear of fœculent matter; he preferred this small dose to the larger ones usually given, and found it to answer the purpose fully.

